# Drying-Induced Structural and Oxidative Transformations in Sustainable Proteins: Impact on Physicochemical Properties and Flavor-Binding Functionality

**DOI:** 10.3390/foods15142478

**Published:** 2026-07-13

**Authors:** Yoon Hlaine Barani, Passakorn Kingwascharapong, Vikas Kumar, Jiaqiang Huang, Shusong Wu, Saroat Rawdkuen

**Affiliations:** 1 Unit of Innovative Food Packaging and Biomaterials, School of Agro-Industry, Mae Fah Luang University, Chiang Rai 57100, Thailand; yoonhlaine288@gmail.com; 2 Department of Fishery Products, Faculty of Fisheries, Kasetsart University, Bangkok 10900, Thailand; passakorn.ki@ku.th; 3 ICAR-Indian Institute of Millets Research, Rajendranagar, Hyderabad 500030, Telangana, India; vkchoprafst@rediffmail.com; 4 Beijing Advanced Innovation Center for Food Nutrition and Human Health, Department of Nutrition and Health, China Agricultural University, Beijing 100083, China; jqhuang@cau.edu.cn; 5 College of Animal Science and Technology, Hunan Agricultural University, Changsha 410128, China; wush688@hunau.edu.cn

**Keywords:** drying modalities, protein-flavor interactions, blue foods, structural reorganization, molecular docking

## Abstract

The rapid global transition toward sustainable food systems has intensified interest in alternative protein ingredients derived from both terrestrial plants and blue foods. However, a critical bottleneck in the commercialization of these proteins is the stabilization of flavor profiles during dehydration. Drying technologies ranging from conventional hot-air and heat pump drying to microwave and vacuum freeze-drying inevitably induce structural reorganization and oxidative modifications. These transformations fundamentally modulate how volatile flavor compounds are bound, retained, and released within the food matrix. This review proposes a comprehensive structure–process–function framework that mechanistically connects intrinsic protein architectures, drying-induced denaturation, and flavor-binding behavior. The review first contrasts globular plant proteins (e.g., soy, pea, and emerging tropical crops) with fibrous marine myofibrillar and collagenous proteins, emphasizing their distinct hierarchies, amino acid compositions, and oxidative vulnerabilities. It then critically evaluates how varying drying modalities drive protein unfolding, aggregation, and carbonylation, and how these transformations alter binding pocket accessibility, surface hydrophobicity, and lipid–protein–flavor crosstalk. Furthermore, it highlights the emerging role of hybrid plant–marine protein matrices as a strategy to optimize techno-functionality. By integrating structural biophysics with computational approaches such as molecular docking and structure-based modeling, this review provides a predictive conceptual map for designing flavor–protein interactions under specific dehydration histories. Ultimately, the proposed framework offers practical design principles for selecting protein sources and tailoring drying strategies to produce high-quality, sensorially superior, and sustainable next-generation food products.

## 1. Introduction

The global demand for sustainable and nutritionally balanced food systems has increased the search for alternative sources of protein from plants and marine sources. Conventional sources of proteins, such as soy, continue to be in use, but the growing environmental concerns and the necessity to diversify protein supply chains have sparked interest in new sources, which include tropical plant proteins and by-products of marine processing [1,2]. These emerging sources offer substantial potential for value-added ingredients and improved resource efficiency in the food industry. In this review, we adopt a structure–process–function framework that links intrinsic protein architecture, drying-induced structural and oxidative changes, and flavor-binding behavior in sustainable protein systems.

Despite their potential, processing operations used for stabilizing and storing protein-based ingredients have a significant impact on their functionality and sensory quality [3]. Drying, a widely used preservation method, extends the shelf life of foods by removing moisture through thermal or non-thermal processes, particularly in the processing of fruits, vegetables, and aquatic products [4]. However, both traditional and advanced drying methods, including freeze-drying, heat pump drying, and microwave-assisted vacuum drying, while effective in lowering water activity, also cause structural changes in proteins [5]. These alterations, including partial unfolding, aggregation, rearrangement of disulfide bonds, and oxidative reactions, may considerably alter the physicochemical characteristics and functional activity of protein matrices. These structural changes are particularly significant as proteins play a critical role in the perception of flavors [6,7]. Volatile compounds interact with protein molecules by hydrophobic interactions, hydrogen bonding, and ligand binding inside internal cavities or on the surface of the protein molecules. However, these binding sites are altered due to structural changes caused by thermal or dehydration reactions, leading to increased retention or faster release of flavor compounds [4]. Therefore, understanding the nature of the correlation between processing-induced structural alterations and protein flavor interactions is vital in the regulation of the sensory properties of dried protein ingredients.

**Figure 1:**
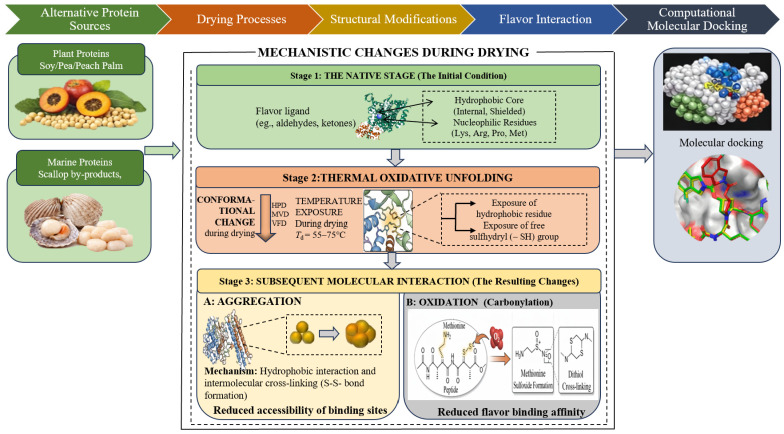
Mechanistic framework of drying-induced structural modifications and flavor-binding dynamics in sustainable protein systems. (Protein structure source: [10]).

An emerging strategy in protein ingredients design involves the strategic integration of multiple biological sources of proteins. Plant proteins are typically globular (storage-rich), whereas marine muscle proteins are predominantly fibrous (contractile-related) [8,9]. The integration of these structurally unique protein systems has recently attracted interest because of the possibilities of cooperative assembly and enhanced functional activity. Hybrid plant–marine protein systems can consequently provide new possibilities to regulate the interaction with volatile substances and increase flavor stability during processing. These systems are a central focus of this review because they illustrate how combining globular plant proteins with fibrous marine proteins creates new opportunities to control drying-induced flavor changes (Figure 1).

In parallel with experimental approaches, computational methods have been increasingly applied to investigate protein–ligand interactions at the molecular level. Methods such as molecular docking and structural modeling enable the prediction of the binding sites of proteins and their relative strengths of interaction with volatile compounds [11]. When combined with experimental values of flavor stability and protein oxidation, these approaches provide deeper mechanistic insights into the factors governing flavor retention in processed protein systems [6].

Several recent reviews have examined sustainable protein ingredients, protein oxidation, drying technologies, or flavor retention individually. These studies have provided valuable insights into specific aspects of protein functionality and properties. However, they have generally focused on isolated topics and have rarely integrated the relationships among protein structure, drying-induced modifications, oxidative transformations, and flavor-binding behavior within a unified framework. Furthermore, comparative discussions between plant and marine proteins remain limited, while hybrid plant–marine protein systems have received relatively little attention.

The present review differs from previous reviews by establishing a structure–process–function framework that links intrinsic protein architecture with drying-induced structural and oxidative changes and their subsequent effects on flavor-binding functionality. Unlike earlier reviews that primarily emphasize processing efficiency or general functional properties, this review provides a mechanistic perspective on how conformational alterations, protein oxidation, and aggregation may influence flavor retention and release. In addition, the review highlights the emerging role of hybrid plant–marine protein systems and discusses the application of molecular docking as a complementary tool for investigating protein–flavor interactions and guiding future research.

## 2. Diversity of Protein Architectures for Sustainable Food Systems

### 2.1. Terrestrial Globular Proteins: Structural Features and Functional Potential

**Figure 2:**
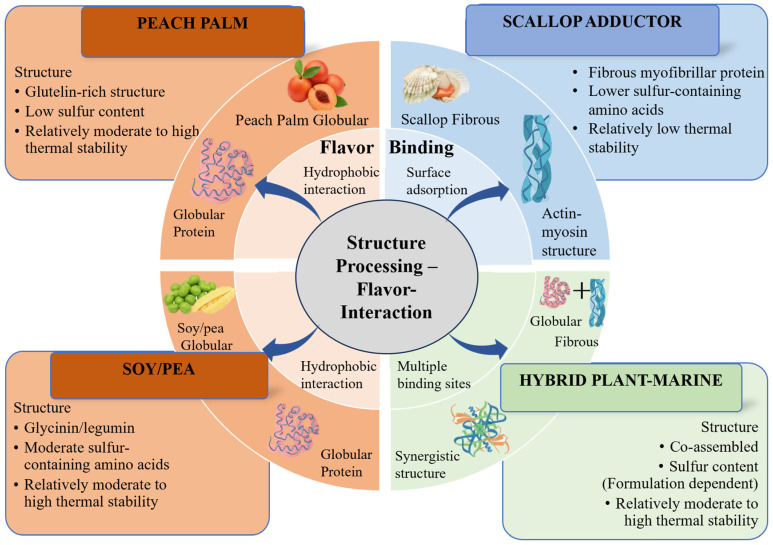
Comparative architectural properties and flavor-binding characteristics of sustainable plant and marine protein systems.

Plant proteins are available as predominantly globular macromolecules which are stabilized by hydrogen bonds and hydrophobic interactions, and disulfide bonds in some cases [12]. These structural characteristics govern both nutritional quality and techno-functional properties in processing, including solubility, emulsification, and flavor binding [13]. The comparative hierarchical architectures of these systems, from fibrous marine muscles to globular plant proteins, are illustrated in Figure 2.

Peach palm (*Bactris gasipaes Kunth*) is an emerging, underutilized tropical crop that represents a sustainable source of plant protein. Peach palm proteins are primarily glutelin-type with dense folded structures rich in hydrophobic amino acids (leucine, valine, phenylalanine), forming inner nonpolar pockets for volatile flavor compounds, though lower total hydrophobicity than soy may reduce binding strength [14]. They contain 23–26 mg sulfur amino acids/g protein (mainly methionine and cysteine), which may increase susceptibility to oxidation and off-flavor development during drying [15]. Lipid content varies (2–14%) across accessions and may influence oxidative pathways during processing [14]. These differences necessitate protein-specific drying strategies to preserve structural integrity and flavor-binding capacity. The relatively lower hydrophobicity of peach palm proteins may impair flavor-binding capacity. Incorporation of marine proteins with higher surface hydrophobicity could potentially provide additional binding regions for volatile compounds; however, the effects of such hybrid systems on flavor retention and flavor-binding behavior require further experimental investigation.

### 2.2. Conventional Legume Proteins as Structural Benchmarks

Soy and pea proteins, which are widely used in the industrial sector and whose functional characteristics are well documented, serve as structural benchmarks in plant-based food systems. These proteins consist mainly of glycinin, 2-conglycinin, and legumin fractions, which form quaternary structures resulting in moderate thermal stability and predictive rheological behavior. Their extensive use has made it possible to develop many products based on plants, but the problem of flavor defects induced by dehydration remains a persistent challenge [16]. Although these proteins are well-characterized and have good functionality, they are not resistant to the loss of flavor during dehydration, which underlines a weakness in their structural strength. The presence of carbohydrates in protein matrices can significantly affect drying stability. Reducing sugars may participate in Maillard reactions with amino groups of proteins, promoting glycation, structural modification, and the generation of undesirable flavor compounds [17]. Carbohydrate-rich matrices also exhibit altered glass-transition behavior and increased stickiness under elevated temperatures, further accelerating protein destabilization during drying [18]. Therefore, the carbohydrate composition of the matrix should be considered when designing drying protocols for protein ingredients.

Thermal dehydration triggers lipid oxidation and unmasks hydrophobic residues, leading to the formation of undesirable beany and grassy off-flavors. The strategic exposure of sulfhydryl and hydrophobic groups can be performed under controlled preheating, enabling the improvement of techno-functionality (foaming and gelling) [19], but the excessive thermal exposure or dehydration causes irreversible unfolding. Even though soy and pea proteins have higher denaturation temperatures (e.g., 90 °C) when hydrated than most of the novel plant proteins, dehydration causes a significant drop in the energetic barrier to the unfolding process, exposing them to oxidative damage [20]. Such well-defined reactions place soy and pea proteins as competitive points of reference for evaluating new protein sources under emerging drying technologies and conditions [21].

### 2.3. Aquatic Fibrous Proteins from Scallop and Marine Processing Residues

Proteins from seafood and scallop processing by-products constitute an architecturally distinct class of biomacromolecules that are predominantly fibrous [8,22]. Myofibrillar proteins, including myosin and actin, as well as collagenous fractions, have elongated forms abundant in α-helical folds and hierarchies. These properties give them good gelling, emulsifying, and water-holding capacities, which are favorable in food structuring applications. Nevertheless, their sensitivity to thermal and oxidative stress is also dictated by these same structural features [23].

Marine proteins are highly susceptible to oxidation because of their high contents of reactive amino acids, endogenous metal ions, and residual lipids. In the process of dehydration, the reactive oxygen species easily cause the formation of carbonyls and backbone fragmentation, which causes aggregation and solubility loss [23]. Unlike globular proteins, fibrous marine proteins possess a unique structural hierarchy dominated by myosin and actin filaments. During drying, these fibrous arrangements are highly susceptible to thermal shrinkage and protein carbonylation, which may alter volatile flavor retention [6].

In terms of flavor retention, fibrous proteins differ from globular proteins in that their hydrophobic groups are distributed along extended chains rather than localized in discrete internal pockets. Although partial unfolding can temporarily facilitate volatile binding, too much denaturation facilitates aggregation and loss of bound aroma compounds [24]. Though it is not directly involved in volatile binding, collagen also affects the perception of flavor indirectly through changing the rigidity of the matrices and the movement of water. All these serve to point out the limited processing window needed to maintain both functionality and sensory quality of marine protein systems. The scallop myofibrillar proteins have sensitive responses to the processing conditions, as demonstrated by their detailed structural characterization. Liu et al. (2022) [25] showed that the best functional characteristics are attained at 200 MPa, and further pressures (>300 MPa) cause irreversible aggregation and a lack of solubility. They are accompanied by quantifiable tertiary structural alterations as evidenced by intrinsic tryptophan fluorescence. These results emphasize that there is a fine line between minimal denaturation (native binding architecture is retained) and enough structural modification (functional gelation is possible), a compromise that is often disrupted by hot air drying but may be preserved by carefully controlled low-temperature dehydration methods (Section 3).

### 2.4. Hybrid Plant–Marine Protein Complexes and Synergistic Binding Effects

Limitations of single-source plant proteins have motivated the development of plant–marine protein systems that combine globular plant proteins with fibrous marine proteins [26]. The above composite systems take advantage of the complementary structural properties of globular and fibrous proteins to form multifunctional scaffolds that are more stable and retain flavor. Defined binding cavities and oxidative buffering capacity may be provided by globular plant proteins and mechanical resilience and network formation by fibrous marine proteins [27].

Electrostatic associations, hydrogen bonding, and hydrophobic interactions at the molecular level determine co-assembled structures in hybrid matrices. Such interactions may lead to altered surface properties and modified ligand-binding behavior, often described as a collapse of the binding pocket [28]. Hybrid systems may contribute to a more uniform distribution of flavor compounds and could potentially improve aroma stability during dehydration. However, the mechanisms underlying the mitigation of localized oxidative reactions in hybrid protein matrices remain insufficiently understood and require further experimental investigation [23,27]. Nevertheless, the effectiveness of these systems is highly dependent on processing conditions; a severe denaturation of either protein fraction may destabilize the inter-protein interactions and reduce the potential synergistic benefits.

### 2.5. Conformational Vulnerability Across Protein Kingdoms

Although protein systems are structurally diverse, all systems are uniformly susceptible to conformational change within dehydrated systems. Other parameters, like denaturation temperature and surface hydrophobicity, can be useful in establishing the stability of proteins in processing stress [29]. Globular plant proteins usually unfold cooperatively, that is, they undergo abrupt changes in surface hydrophobicity above a critical temperature or moisture level. Conversely, fibrous marine proteins experience a more gradual loss of structure, which is associated with the progressive disorganization of the ordered areas of a protein backbone.

### 2.6. Implications for Sustainable Protein Processing

The variety of protein structures that can be traced to land and water sources requires moving beyond homogeneous processing strategies. Variations in amino acid composition, folding patterns, and conformational fragility have a direct effect on the dehydration response of proteins and their capacity to preserve flavor molecules [16]. Understanding these differences allows the rational choice of the protein sources and the adaptation of drying technologies to the particular molecular structures. Combining detailed structural knowledge with precision dehydration can optimize the functional and sensory attributes of sustainable protein ingredients. The marine by-product proteins also have unique amino acid content and structural flexibility as compared to the traditional legume proteins, and could have a substantial impact on interaction with volatile compounds. These discrepancies underscore the need for protein-architecture-specific processing strategies that optimize functional performance and flavor stability.

## 3. Advanced Drying Methodologies: Mechanics and Structural Implications

**Figure 3:**
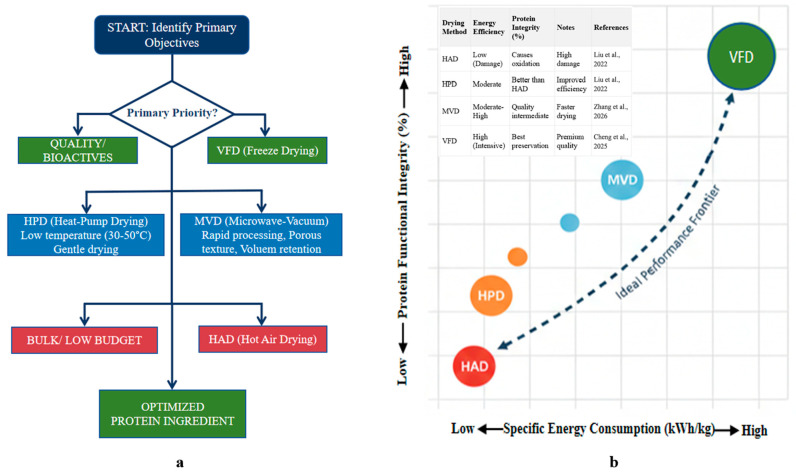
Conceptual framework (**a**) and quality plot (**b**) between energy consumption and functional integrity of protein [4,5,30].

Drying technologies play a critical role in stabilizing protein ingredients by reducing the water activity (aw), thereby improving long-term storage stability [4]. However, dehydration inherently induces alterations in protein conformation and oxidation states, which subsequently influence protein–flavor interactions. Various drying methods have different thermal and mass-transfer requirements, which dictate the degree of structural change [30]. Among the emerging technologies, heat pump dehydration (HPD), microwave vacuum drying (MVD), and vacuum freeze-drying (VFD) have been extensively evaluated as strategic balances between product quality and processing efficiency (Figure 3).

Globular and fibrous proteins exhibit different structural responses during drying due to their distinct molecular architectures. In globular proteins such as soy proteins, thermal drying typically promotes partial unfolding, accompanied by reductions in α-helical content and increases in β-sheet structures. These results lead to altered surface hydrophobicity and flavor-binding behavior [14,21]. In contrast, fibrous marine proteins such as myofibrillar proteins undergo filament shrinkage, aggregation, and cross-linking during drying, which reduces solubility and disrupts volatile binding regions. These architecture-dependent structural responses underline the importance of protein-specific drying strategies [5]. The oxidative consequences of these structural changes, including carbonylation and sulfhydryl loss, are discussed in detail in Section 4.

### 3.1. Heat Pump Dehydration (HPD): Low-Temperature Moisture Removal and Surface Integrity

Heat pump dehydration (HPD) is a promising low-energy substitute for hot air drying of heat-sensitive food matrices, including protein-based food systems. HPD uses a closed-loop system with dehumidified, recirculated air and heat recovery, unlike conventional convective dryers. The moist air from the drying chamber passes through an evaporator where water vapor condenses, and the latent heat is recovered via a condenser, allowing precise control of temperature and relative humidity. This design enables HPD to operate at temperatures typically around 35–55 °C, imposing minimal thermal stress on protein matrices [4].

The primary physicochemical advantage of HPD is the separation of moisture removal and uncontrolled surface heating. In conventional hot air drying, evaporation rapidly occurs at the surface, and the result is commonly a dense, dry crust, the so-called case hardening. This resists the penetration of internal moisture and prolongs drying time. It also increases internal thermal gradients. In HPD, case hardening is alleviated by maintaining a moderate vapor pressure gradient between the product surface and the surrounding air, allowing more uniform moisture migration from the interior to the surface. The lower oxygen availability and reduced surface temperature further limit ROS formation, thereby preserving protein secondary and tertiary structures [31,32].

Empirical comparisons between HPD and other drying technologies in scallop adductor muscle provide direct evidence for these structure-preserving effects. Liu et al. (2023) [4] compared hot air drying (HAD), HPD, and vacuum freeze-drying (VFD) for *Patinopecten yessoensis* adductors and reported that HPD produced a looser microstructure and less severe degradation of protein secondary structure than HAD, although VFD maintained the most porous structure and highest rehydration capacity. Low-field NMR data showed that HPD, like VFD, preserved immobilized water populations more effectively than HAD, indicating less extensive protein network collapse and better water-holding potential in the dried scallop. In a related study on golden pompano (*Trachinotus ovatus*), heat pump drying generated markedly lower losses of total sulfhydryl groups and less increase in surface hydrophobicity than hot air drying [32].

For plant proteins, recent work on soy and pea isolates has clarified how thermal history and drying conditions govern unfolding, aggregation, and functional properties. More intense thermal treatments promote larger, less soluble aggregates [21,33]. Similar behavior is observed for soy protein isolates, where preparative heat treatments drive aggregate formation that stiffens heat-induced gels but reduces solubility and toughness [34]. In the context of peach palm, compositional analyses highlight a high content of storage proteins and lipophilic bioactive compounds that are susceptible to thermal and oxidative degradation, underscoring the need for gentle dehydration strategies to retain functionality and flavor [14]. Collectively, these findings indicate that low-temperature, humidity-controlled HPD environments are effective in preserving protein structure and limiting excessive aggregation, though HPD does not fully preserve native protein architecture to the same extent as freeze-drying.

### 3.2. Microwave Vacuum Drying (MVD): Volumetric Heating and Thermal History Reduction

Microwave vacuum drying (MVD) represents a fundamentally different approach to dehydration, combining electromagnetic energy with reduced-pressure environments to achieve rapid moisture removal at relatively low effective temperatures. In MVD, microwave energy penetrates the food matrix and induces volumetric heating through dipole rotation and ionic conduction, while vacuum conditions reduce the boiling point of water, facilitating evaporation at temperatures often below 40–50 °C. The key advantage of MVD for protein preservation lies in its ability to minimize the “thermal history” of the product. Thermal history encompasses not only peak temperature but also the duration of thermal exposure, both of which strongly influence protein denaturation, aggregation, and oxidation. By accelerating internal moisture removal and shortening drying time by up to 30–50% compared with convective drying, MVD substantially reduces the opportunity for ROS generation and oxidative chain reactions [35].

Structurally, volumetric heating reduces localized surface overheating, promoting a more uniform temperature distribution throughout denser protein matrices where convective heat transfer is inefficient. In the case of globular plant proteins such as soy and pea isolates, a reduced thermal history inhibits cooperative unfolding events that would otherwise expose hydrophobic residues [5]. Bhambhani et al. (2021) [36] reported that MVD reduced drying time by approximately 87% (6.5 h vs. 50 h for lyophilization) while achieving similar catalase activity retention (78.3% for MVD vs. 80.9% for freeze-drying), indicating that substantial reductions in drying time are possible without loss of protein function. MVD has also been shown to preserve volatile compounds more effectively than hot air drying and, in some cases, to approach freeze-drying in flavor retention at considerably lower energy cost.

A notable challenge of MVD is the potential for localized hot spots in systems with heterogeneous dielectric properties or uneven moisture distribution, which can lead to non-uniform denaturation and structural collapse [5]. Careful regulation of microwave power, vacuum level, and sample geometry is therefore required to balance drying efficiency with molecular preservation. Scaling and homogeneity remain non-trivial challenges when protein mixtures are compositionally heterogeneous.

### 3.3. Vacuum Freeze-Drying (VFD): Sublimation and Structural Preservation

Vacuum freeze-drying (VFD) is the reference technology for preserving structural and sensory attributes of thermally sensitive foods. The process involves freezing the product, then sublimating ice under low pressure so that water is removed without passing through the liquid phase. This mass-transfer pathway minimizes capillary stress and structural collapse, producing highly porous matrices that closely resemble the original microstructure [37].

VFD provides excellent preservation of protein secondary and tertiary structures: molecular mobility is immobilized during freezing, which suppresses oxidation reactions and protein unfolding, thereby maintaining volatile-binding pockets [5]. Comparative research on whey protein concentrates, lysozyme, and myoglobin suggests that freeze-dried powders tend to retain near-native secondary structure and monomer content, with equivalent or less aggregation than spray-dried counterparts. The preservation of whey protein structure during spray drying also depends on the choice of carrier agent and the control of foam formation. Appropriate drying aids such as maltodextrin or gum arabic can protect protein structure during atomization, while excessive foam formation may increase protein exposure at air–water interfaces, promoting unfolding and functional loss during drying [38,39]. Freeze-drying of golden pompano filets and tilapia muscle in marine systems causes more intact myofibrils, reduced myoglobin oxidation, and improved color and texture compared to all other drying methods [38]. VFD’s porous microstructure excels in flavor entrapment and rehydration but is limited by high energy, long times, and capital costs, prompting alternatives like HPD/MVD that approximate its benefits more scalably [5]. Although freeze-drying generally preserves the native secondary and tertiary structure of globular proteins, flavor quality may still be affected by Maillard reactions between residual reducing sugars and free amino groups [17,40]. This means that structural stability and flavor stability do not necessarily occur simultaneously: a protein may retain its folded conformation following lyophilization while generating flavor-active compounds during storage or subsequent thermal exposure.

### 3.4. Comparative Kinetic Modeling: Effective Moisture Diffusivity and Protein Structural Response

Kinetic studies provide a quantitative understanding of the effects of various dehydration technologies on protein structure and functionality. Effective moisture diffusivity (*Deff*)—the integrated result of diffusion, capillary movement, and vapor transport—depends strongly on temperature, pressure, and matrix structure [41]. Drying processes differ markedly in their effective moisture diffusivity (*Deff*) values. In HPD, the relatively low but consistent diffusivity is achieved by moderate temperatures (≈35–55 °C) and controlled humidity, which allows uniform drying and removes as much structural stress as possible [42]. MVD greatly enhances *Deff* through volumetric heating and vacuum conditions, enabling rapid moisture elimination, though high moisture and temperature gradients may occur if power density is not carefully managed. Freeze-drying proceeds via sublimation rather than conventional diffusion, making standard diffusion models less applicable, and provides superior structural preservation despite lower effective diffusivity [4].

Diffusivity and moisture content are closely related to protein unfolding and aggregation. Since water is lost, hydrogen-bonding networks that stabilize protein structures are disrupted, lowering the energy barrier to conformational change. Large internal moisture gradients, typical of high-temperature convective drying, accelerate this process. By contrast, moderate driving forces in MVD and HPD reduce the overall thermal history relative to hot air drying, provided that localized hot spots and excessive mechanical forces are avoided [5,43].

Combining kinetic modeling with molecular indicators, including surface hydrophobicity, carbonyl content (discussed in detail in Section 4), and secondary structure composition, enables macroscopic drying curves to be related to microscopic structure–function relationships. Studies on heat-pump-dried kelp, *Clanis bilineata* protein, and golden pompano muscle consistently show that extreme, high-rate drying conditions worsen functional properties, whereas optimized HPD/MVD preserves solubility, emulsifying behavior, and gelation capacity [43,44]. These findings emphasize that optimal drying cannot be achieved by simply maximizing *Deff*; rather, drying kinetics must be calibrated to the conformational sensitivity of the specific protein system to achieve the desired balance between process efficiency and molecular performance.

### 3.5. Energy Efficiency Versus Quality: Techno-Economic Considerations

Selecting an optimal drying technology for sustainable protein systems necessitates a strategic equilibrium between product quality, energy consumption, processing duration, and financial viability. Conventional hot air drying offers low capital cost and operational simplicity, but its elevated temperatures and prolonged residence times produce adverse effects on color, flavor, and protein functionality that limit its use for high-value protein ingredients [4]. VFD remains the gold standard for quality preservation but is constrained by energy consumption and low throughput. HPD and MVD emerge as intermediate solutions, minimizing energy requirements without substantially compromising sensory and functional characteristics. Recent life-cycle assessments indicate that HPD can achieve 30–50% energy savings relative to hot air drying, while MVD provides significant time savings and reduced cumulative thermal exposure [4,5].

Beyond energy and speed, emerging evidence emphasizes that selecting a drying technology must prioritize the preservation of protein tertiary structures and functional binding sites [32]. By retaining native molecular attributes, including hydrophobic cavities and reactive thiol groups that govern flavor-binding dynamics (see Section 4), advanced methods such as HPD and MVD reduce the need for post-processing interventions such as flavor masking or reformulation. However, their efficacy depends strictly on process optimization; excessive thermal or mechanical stress can negate any structural advantages. As a result, a comprehensive approach to designing drying protocols will require integration of the structural stability of proteins during processing, the kinetic behavior of drying processes, and desired sensory attributes to optimize both cost-effectiveness and sustainability of the final protein products.

Advanced drying methods, including HPD, VFD, and MVD, provide significant improvements over traditional methods by minimizing thermally induced structural damage. However, the extent to which these advanced drying methods reduce oxidative damage is highly dependent on the level of process optimization achieved and the heterogeneity of the dried protein materials. For example, MVD can produce localized overheating in multi-component systems with heterogeneous dielectric properties, leading to inconsistent structural preservation. Similarly, HPD under suboptimal humidity conditions may permit slow conformational degradation over prolonged drying times [45]. Variability across studies in raw materials, pretreatments, and analytical methodology further complicates cross-study comparisons and underscores the need for unified assessment frameworks that incorporate molecular, structural, and sensory measures. Table 1 provides a comparative summary of drying techniques with respect to structural integrity preservation across terrestrial and marine protein systems.

## 4. Oxidative Modification and Flavor-Binding Dynamics

**Figure 4:**
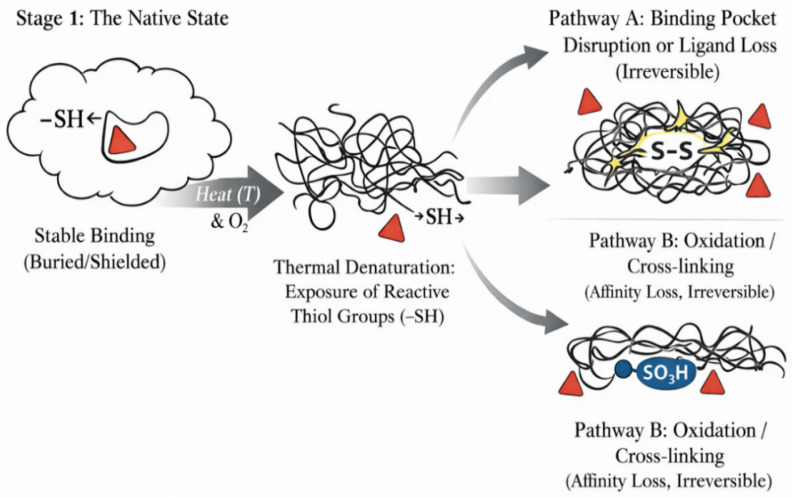
The “Pocket Deletion” Model: Mechanistic pathways of drying-induced flavor instability. Red triangles represent flavor ligands; yellow highlights indicate disulfide bond formation (S–S), and the blue circle (SO_3_H) denotes oxidized thiol groups.

While Section 3 describes how drying methods differ in their structural outcomes, this section addresses the underlying oxidative chemistry that links dehydration conditions to flavor-binding behavior. Dehydration exposes proteins to ROS such as hydroxyl radicals, superoxide anions, and hydrogen peroxide, triggering carbonylation, a prevalent irreversible modification linked to functionality loss and flavor-binding disruption [53] (Figure 4). Oxidation of susceptible amino acid side chains, particularly lysine, arginine, proline, and threonine, is a primary source of protein carbonylation, which is further amplified by reactions with lipid-derived aldehydes, such as malondialdehyde and 4-hydroxy-2-nonenal. The addition of carbonyl groups changes the charge distribution and steric structure of the polypeptide chain, disrupts intramolecular hydrogen bonding, and destabilizes tertiary structure [4].

Proteins interact with flavor compounds via hydrophobic interactions, van der Waals interactions, hydrogen bonding, and in some cases, electrostatic interactions. These are very localized in folded regions of the protein where the nonpolar amino acid residues form microenvironments conducive to the preservation of volatile compounds [54]. Oxidative unfolding may also temporarily increase surface hydrophobicity, which may increase the short-term adsorption of some volatiles. However, prolonged oxidation can promote protein aggregation and reduce flavor retention.

In lipid-rich protein matrices such as peach palm protein isolates, flavor deterioration is further complicated by lipid–protein interactions. Lipid peroxidation generates reactive carbonyl species, including malondialdehyde and 4-hydroxy-2-nonenal, that accelerate protein carbonylation and simultaneously produce off-flavor volatiles such as aldehydes and ketones. This coupled lipid–protein–flavor deterioration pathway results in both loss of desirable aroma retention and accumulation of undesirable oxidation-derived volatiles [55].

### 4.1. Quantitative Evidence of Protein Carbonylation Across Drying Modalities

Recent quantitative research highlights how different drying methods dictate the extent of molecular damage in proteins. Chen et al. (2022) [32] demonstrated the protein carbonyl content of fish myofibrillar proteins under four different drying conditions, revealing a clear sequence of oxidative damage. Hot air drying increased the protein carbonyl content to 2.17 nmol/g protein (5.9 times higher than the 0.37 nmol/g protein of fresh material), while heat pump drying reduced it to 1.04 nmol/g (an increase of 2.8 times). Vacuum freeze-drying resulted in the least damage, with a content of 0.52 nmol/g protein (an increase of 1.4 times). Higher carbonyl levels were also associated with deterioration of functional properties. FTIR spectroscopy revealed pronounced α-helix-to-β-sheet transitions in hot-air-dried samples, indicative of protein unfolding and aggregation, whereas HPD and VFD samples retained substantially greater α-helical content [32].

Elevated protein carbonyl levels have been associated with severe reduction in water holding capacity in model myofibrillar systems subjected to carbonylation-inducing conditions (e.g., glucose, methylglyoxal, or lipid-derived aldehydes), demonstrating that carbonylation is not just an analytical marker but a direct cause of functional collapse [53,56]. These results indicate that protein oxidation can negatively affect structural stability and flavor-binding properties. A comparative summary of quantitative oxidation markers and structural changes across different protein systems and drying methods is provided in Table 2, highlighting the relationship between carbonyl content, sulfhydryl loss, secondary structure alteration, and volatile retention.

### 4.2. Mechanisms of Thermal-Oxidative Crosstalk

Thermal dehydration can enhance interactions between protein oxidation and lipid oxidation by increasing molecular mobility and destabilizing the hydration shell that normally protects proteins. Dehydration removes protective water layers around amino acid side chains and creates additional metal-binding sites, increasing susceptibility to oxidative reactions [9]. Proteins from marine sources, such as scallop myofibrillar proteins and collagen, contain high levels of the transition metals (iron and copper), which can enhance ROS formation via Fenton-type reactions, thereby increasing susceptibility to oxidative cross-linking. These long, fibrous proteins are especially susceptible to oxidation-induced crosslinking, thus resulting in reduced solubility, impaired gelation, and diminished flavor-binding abilities [30]. Overall, oxidative vulnerability is composition-specific, as well as a markedly protein architecture–process environment-dependent characteristic.

### 4.3. Sensory Implications and Structural Synthesis

The extent of oxidative modification is primarily determined by the drying method and the thermal conditions it imposes, a relationship whose structural basis was quantified in Section 3 and Section 4.1. Hot air drying, with its high temperatures and prolonged oxygen exposure, promotes large-scale carbonylation and aggregation. Advanced methods such as HPD and MVD reduce oxidative damage through decreased oxygen exposure, lower effective processing temperatures, and shorter drying times, while VFD provides the greatest protection by operating at low temperatures under near-anaerobic conditions, albeit at a high energy cost [9].

From a sensory perspective, oxidative modification can be observed as the loss of typical aroma sensation and the development of off-flavors commonly denoted as being beany, metallic, or cardboard-like. These defects are not caused only by the production of new oxidation-derived volatiles (e.g., Strecker aldehydes, lipid-derived aldehydes, and ketones) but also by the fact that the protein matrix lost the capacity to hold and release desirable flavor compounds gradually as binding pockets were destroyed or removed [54]. Preserving flavor quality therefore requires an integrated understanding of protein oxidation, structural change, and flavor-binding capacity under different drying conditions, as detailed in Section 3 and Section 4.

The interaction between the drying-induced thermodynamic changes and protein structural responses is systematically presented in Table 1 and Table 2. Although Table 1 summarizes the impacts of various drying modalities on structural integrity in both terrestrial and marine protein systems, demonstrating that lower-temperature, reduced-oxygen drying consistently produces less severe secondary structure disruption and better preservation of hydrophobic binding regions. Table 2 extends this by consolidating quantitative oxidation markers and flavor-binding data across marine and plant protein systems, enabling direct cross-system comparison of how processing-induced structural changes translate into measurable differences in volatile retention and binding capacity.

Together, Table 1, Table 2 and Table 3 demonstrate that flavor retention is closely coupled to the preservation of native protein structure, and that protein-specific differences in oxidative sensitivity mean no single drying protocol is universally optimal across marine and plant systems. Computational molecular docking provides a complementary tool for interpreting these structure–function relationships at the molecular scale, as discussed in Section 5.

## 5. Computational Molecular Docking: The In Silico Interface

Computational molecular docking is an increasingly used tool for studying molecular interactions in complex food systems [60]. In contrast to traditional experimental methods, which mainly quantify a macroscopic end point such as flavor retention or volatile release, docking techniques provide the possibility to study the molecular level of interaction of proteins with their ligands. These methods allow identifying the key structural determinants of flavor-binding capacity because they predict desired binding orientations and relative energies of protein–aroma compound interactions [61].

Docking analysis can be useful in the context of food protein oxidation and processing to assess how the accessibility and stability of hydrophobic binding pockets can be affected by conformational changes. These types of computations thus serve as an addition to an experimental method of analysis by providing a structural framework through which it may be possible to interpret observed changes in flavor retention after drying, oxidation, or other types of processing treatment.

### 5.1. Binding Affinity Simulations and Thermodynamic Interpretation

Molecular docking is used to predict the interaction between a protein and a flavor ligand by estimating the Gibbs free energy change (ΔG) of binding. Ligands that are common in the context of protein-based food are usually esters, aldehydes, and ketones, which provide fruity, green, and fatty aromas [59]. A combination of hydrophobic interactions, van der Waals forces, hydrogen bonding, and steric complementarity between the ligand and the protein binding site controls binding affinity. In docking studies, lower values of ΔG (or lower docking scores, depending on the scoring function used) represent more favorable interactions and are often used to rank ligands by their predicted binding strength. Docking scores, however, are relative estimates rather than absolute thermodynamic quantities and should therefore be interpreted with caution and always used in conjunction with experimental data.

Several studies have demonstrated the usefulness of molecular docking for investigating flavor–protein interactions in food systems. For example, Snel et al. [59] reported through flavor partitioning modeling that interactions between ketone and ester flavor compounds and plant proteins varied according to both protein source and flavor structure. These studies provide supporting experimental evidence that protein conformation plays an important role in flavor retention. Similarly, Sun et al. [62] combined molecular docking with spectroscopic analyses to investigate interactions between spice-derived flavor compounds and myofibrillar proteins, demonstrating that hydrophobic interactions and hydrogen bonding contributed significantly to flavor binding. These studies illustrate how molecular docking can complement experimental observations by providing molecular-level insights into protein–flavor interactions.

More recent docking studies have provided quantitative binding data for specific plant protein–flavor systems. Yang et al. (2026) [63] investigated the interaction of hexanal, the primary beany off-flavor compound in soy-based products, with soy protein isolate (SPI) using molecular docking combined with HS-SPME-GC-MS and circular dichroism analyses. Three binding modes were identified: a hydrophobic cavity, a groove located between α-helix and β-sheet regions, and surface-exposed sites. The hydrophobic cavity represented the most energetically favorable binding configuration, with a predicted binding energy of −4.5 kcal/mol, indicating that nonpolar amino acid residues lining the cavity create a preferential environment for aldehyde accommodation. Similarly, Ince et al. (2025) [64] applied blind docking with AutoDock Vina (v 1.1.2) to characterize hexanal binding to soy 11S glycinin, reporting a binding energy of −5.8 kcal/mol with interactions localized in the hydrophobic calyx region of the protein subunit, confirmed by fluorescence quenching analysis yielding association constants (KA) ranging from 3.1 × 10^2^ to 3.1 × 10^4^ M^−1^. For pea protein isolate, Bi et al. (2022) [58] demonstrated that flavor compound molecular structure critically determines binding mode and affinity: hexanal interactions were dominated by hydrogen bonding (K ≈ 684 M^−1^), whereas the more hydrophobic and unsaturated (E)-2-octenal exhibited substantially stronger binding through hydrophobic interactions (K ≈ 8208 M^−1^), with molecular docking confirming distinct binding regions within the pea protein tertiary structure (PDB: 3KSC). Collectively, these findings highlight that both protein architecture and flavor compound physicochemical properties, particularly hydrophobicity, chain length, and degree of unsaturation, govern binding site selection and interaction strength in plant protein systems. Notably, the predicted docking binding energies for hexanal in soy protein systems (approximately −4.5 to −4.7 kcal/mol) suggest moderate predicted affinity, although direct cross-study comparison should be interpreted cautiously because docking scores depend on the model, software, and system conditions [63].

Oxidative changes, particularly protein carbonylation and reactions with lipid-derived aldehydes, can directly diminish binding capacity by altering charge distribution and surface hydrophobicity [53]. Such oxidation-driven structural shifts have been experimentally confirmed in myofibrillar protein systems, where treatment with lipid oxidation products caused measurable increases in surface hydrophobicity and secondary structure rearrangement under thermal processing conditions, as discussed in detail below.

Thermodynamic arguments also suggest that ligand accommodation may be enhanced by moderate conformational flexibility, whereas excessive unfolding or aggregation may disrupt or occlude binding regions and limit effective ligand binding. This is one of the reasons why mild processing may enhance short-term aroma adsorption, whereas severe dehydration and oxidation may be linked to accelerated aroma loss and poorer controlled release [13]. Therefore, docking-derived affinity scores are best used when considered in combination with processing history (thermal exposure, oxygen availability) and the structural/oxidation biomarkers in Section 3 and Section 4.

Docking principles have also been extended to receptor-based systems, as illustrated by Zhu et al. (2021) [65], who screened cod-derived umami hexapeptides against a T1R1/T1R3 homology model. Docking results showed that INKPGL exhibited the lowest binding energy (−109.1 kcal/mol, CDOCKER), indicating the highest predicted umami affinity among the five hexapeptides tested, with key interactions at residues S104, S146, and D249, confirmed by electronic tongue and sensory evaluation, demonstrating that docking predictions can successfully guide identification of bioactive peptides from marine protein sources [65].

Beyond taste receptor interactions, molecular docking has also been applied to investigate the binding of lipid oxidation products—key drivers of off-flavor formation to myofibrillar proteins under thermal processing conditions. Shen et al. (2022) [56] examined the interaction of malondialdehyde (MDA) and 4-hydroxy-2-nonenal (HNE) with sturgeon myofibrillar protein (myosin, PDB: 3QMA) using AutoDock Vina combined with isothermal titration calorimetry (ITC), FTIR, and fluorescence spectroscopy. Docking predicted binding energies of −5.5 kcal/mol for MDA and −4.1 kcal/mol for HNE, with MDA forming six hydrogen bonds at residues HIS-78, LEU-71, and LEU-72, and HNE forming three hydrogen bonds primarily at LYS-67. ITC measurements confirmed these predictions, yielding negative ΔG values across all treatment temperatures (50–70 °C), with KD values decreasing as temperature increased, indicating that thermal treatment progressively enhanced binding affinity between lipid oxidation products and myofibrillar protein. Importantly, the stronger predicted binding of MDA relative to HNE (−5.5 vs. −4.1 kcal/mol) was consistent with ITC-derived thermodynamic data, representing one of the few examples in the current literature where docking predictions have been directly validated by calorimetric measurements in a food protein system under realistic processing conditions. Furthermore, conformational analyses revealed that both MDA and HNE treatments caused a significant decrease in α-helix content and an increase in β-sheet content, accompanied by increased surface hydrophobicity, structural changes consistent with protein unfolding that may progressively alter the accessibility of hydrophobic binding regions for aroma compounds. These findings are particularly relevant to understanding how drying-induced oxidation of myofibrillar proteins may shift binding behavior from reversible noncovalent interactions toward covalent modification, thereby contributing to off-flavor development and reduced aroma retention in processed sustainable protein ingredients (Table 4).

Recent reviews of protein–flavor interactions distinguish reversible physical binding (van der Waals forces, hydrogen bonds, hydrophobic association) from irreversible covalent binding driven by reactive lipid-oxidation aldehydes reacting with nucleophilic residues, supporting a ‘reversible vs. irreversible retention’ conceptual framework. For sustainable protein design, maintaining predominantly reversible binding architectures during dehydration is critical because irreversible covalent modification is associated with off-flavor formation and long-term sensory deterioration [56].

### 5.2. Structural Visualization of Flavor-Binding Pockets and Oxidation-Induced Accessibility Changes

In addition to numerical docking scores discussed in Section 5.1, molecular docking provides three-dimensional visualization of binding poses and pocket environments, allowing interpretation of how processing-induced structural changes affect ligand accommodation. In protein–flavor systems, the geometry and chemistry of hydrophobic regions and hydrogen-bond networks in folded domains, as revealed by docking, help map ligand orientation, residue contacts, and (when coupled with pocket-mapping tools) changes in pocket accessibility and volume [44].

As discussed in Section 4, oxidative modification may alter the accessibility of flavor-binding regions. In the present review, the term “binding pocket disruption or occlusion” is used here as a conceptual hypothesis to describe the potential reduction in accessibility of hydrophobic binding regions resulting from oxidation-induced structural changes. Experimental studies have demonstrated that oxidation can influence protein conformation, surface hydrophobicity, and flavor-binding behavior. However, direct experimental evidence demonstrating actual binding-pocket collapse or occlusion remains limited. Therefore, this concept should be regarded as a mechanistic hypothesis that may help explain reductions in flavor retention following severe drying and oxidation, but it requires further experimental validation [66,67].

While docking provides valuable thermodynamic and structural insights into protein–flavor interactions, quantitative comparisons between native and oxidatively modified protein structures remain limited. Structural visualization of binding pockets, combined with experimental data on oxidation and conformational changes, may help explain how processing-induced structural rearrangements alter the accessibility of potential binding regions and thereby affect protein–flavor interactions [16,62,68,69,70].

### 5.3. Perspective on Predictive Design

Molecular docking provides a useful tool for predicting the influence of protein structure and oxidative modification on flavor-binding behavior; however, it remains a predictive approach that requires experimental validation in sustainable protein systems. One major limitation is that docking simulations are generally based on static protein structures and simplified scoring functions, which may not fully capture the dynamic and heterogeneous nature of food matrices [60,61]. Furthermore, relatively few studies have directly compared ligand-binding behavior between native and oxidatively modified proteins, limiting the current understanding of oxidation-induced changes in flavor retention. Most docking models do not adequately account for protein conformational flexibility, solvent effects, or interactions with other food components such as lipids and polysaccharides. Consequently, docking-derived affinity values should be interpreted cautiously and validated experimentally whenever possible.

To improve reliability and practical relevance, future studies should combine docking predictions with experimental approaches such as HS-GC-IMS, GC-MS, FTIR, and differential scanning calorimetry (DSC) [6,67]. Multi-scale validation across different protein systems, including plant, marine, and hybrid matrices, would enable a more robust evaluation of the relationship between predicted binding affinities and experimentally measured flavor retention [13,56]. In this context, molecular docking can serve as a complementary tool for guiding the rational design of sustainable protein ingredients with improved flavor stability under drying and oxidative conditions. Future integration of molecular dynamics simulations with analytical techniques such as HS-GC-IMS and GC-MS may further improve the predictive value of computational approaches and strengthen their application in flavor-retention studies. Furthermore, advanced non-thermal processing technologies, including ultrasound, cold plasma, pulsed electric field, ozone treatment, and ultraviolet irradiation, represent promising future research directions for reducing off-flavor compounds and modifying protein structure in sustainable protein ingredients, particularly for plant-based systems, and warrant dedicated investigation in future studies.

## 6. Experimental Validation and Translational Integration of Flavor–Protein Stability

Experimental validation of protein–volatile interaction hypotheses, particularly docking-derived binding poses, requires sensitive analytical methods for volatile organic compound (VOC) analysis. Among these methods, HS-GC-IMS has become widely used because it can detect VOCs at very low concentrations in complex food matrices and identify processing-induced changes in aroma composition [67]. HS-GC-IMS can generate characteristic volatile profiles that may be correlated with protein structural indicators such as carbonyl content and FTIR spectra [71]. This approach can also be used to determine whether advanced dehydration methods that preserve native protein conformation can effectively maintain volatile compounds during processing [24]. As docking is dependent on methodology, the docking scores should therefore be treated as relative indicators. One practical strategy is to use molecular docking to identify potential high-affinity ligands, followed by HS-GC-IMS combined with chemometric analyses such as PCA and PLS-DA to evaluate whether stronger predicted interactions are associated with reduced flavor loss [72,73].

### 6.1. Synergistic Framework: Bridging Macro-Volatile Profiles with Micro-Molecular Docking

**Figure 5:**
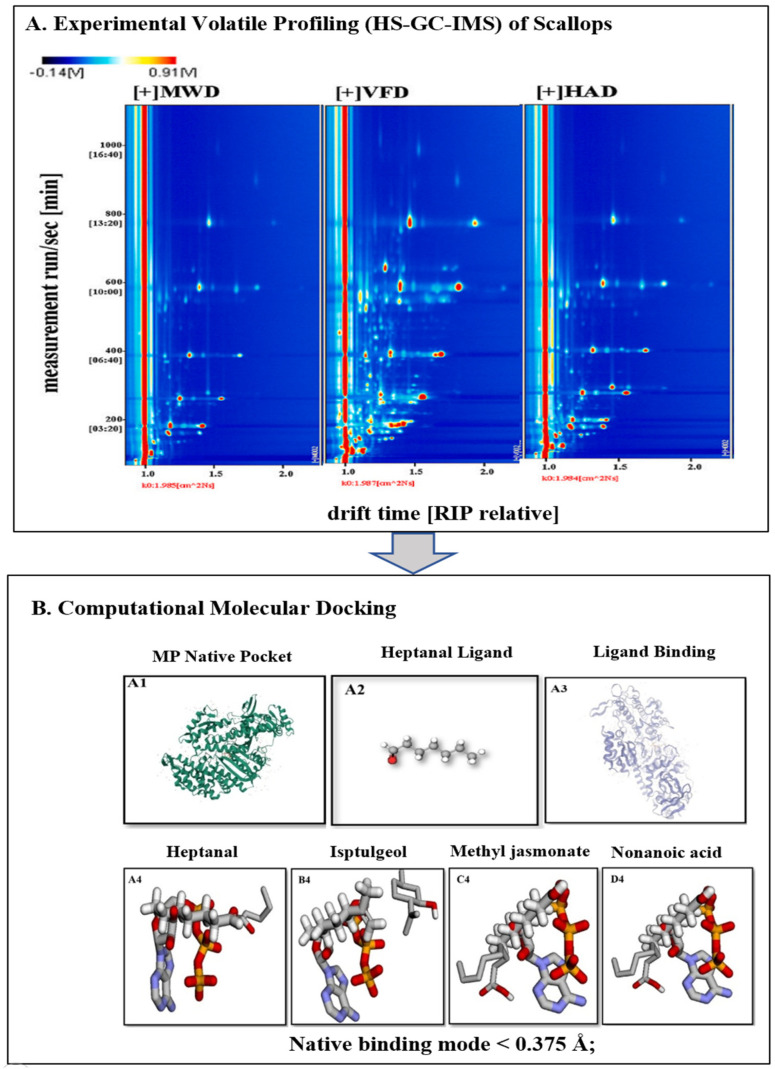
Synergistic integration of (**A**) experimental HS-GC-IMS volatile profiling of dried scallops (sourced from [52]), (**B**) in silico molecular docking of myofibrillar protein (MP) and flavor compounds. Protein (A1), Heptanal (A2), Isptulegol, Methyl jasmonate, Nonanoic acid; 3D docking mode between protein and Heptanal (A3), Isptulegol, Methyl jasmonate, Nonanoic acid; the detailed 3D binding mode of the myosin complex (distance < 3.5 Å; Heptanal (A4), Isptulegol (B4), Methyl jasmonate (C4), Nonanoic acid (D4).

To bridge the critical gap between macroscopic volatile release and microscopic protein interactions, a synergistic analytical framework is proposed, as illustrated in Figure 5. The multiscale correlations confirm that the protein structures resulting from processing will enable the stable function of the flavor–protein complex.

Using this framework, the spatial and intensity of flavors as visualized in the 2D topographical vertical view of HS-GC-IMS (Figure 5A) clearly illustrate the impact of different drying methods on the scallop (*Patinopecten yessoensis*) adductor muscle. Notably, the significant flavor deviations observed in the HAD samples were significantly different from those in the VFD, indicating the need for low-thermal processing for fragile marine matrices [52].

The experimental findings described above are supported by computational molecular docking studies (Figure 5B), in which key ligands such as Heptanal and 2-Nonanone bind to myofibrillar proteins (MPs) (distance < 0.375 Å), as indicated by high-affinity binding modes. The results provide additional molecular-level evidence explaining the flavor retention trends seen in chromatography data. Research has recently demonstrated that proteins that have a higher predicted binding affinity to ester and aldehyde ligands will retain a greater percentage of volatiles after dehydration, while proteins that are oxidized or denatured will have a lower amount of volatile loss, but will also display simpler fingerprints [44,74]. Additionally, using multivariate statistical techniques allows molecular docking to be transformed from a qualitative visualization into a semi-quantitative predictive tool for determining flavor stability, providing a solid base for optimization of sustainable protein-based food products.

### 6.2. Matrix Effects and the Limits of In Silico Simplification

The docking simulation is an extremely useful tool for understanding how flavors bind to foods. Docking simulations provide valuable mechanistic detail regarding how food flavors bind to food systems, but this is achieved through simplifying many aspects of the food system. For example, in real-world food systems, food-matrix proteins exist in a variety of microenvironments, such as those influenced by water activity, ionic strength, and polysaccharides, lipids, and processing-induced alterations in microstructure. These factors impact protein structure, aggregation properties, and volatile distribution. Therefore, in silico predictions could be viewed as idealized estimates generated under controlled conditions rather than an accurate representation of what happens in a complex food matrix [75]. Experimental research indicates that proteins with equivalent docking affinities can exhibit vastly different volatile retention profiles depending on the overall composition of the matrix. In marine myofibrillar proteins, high predicted aldehyde content is rapidly lost upon aggregation that occurs from the onset of dehydration, thereby disrupting volatile entrapment despite molecular-level affinity. Moreover, external variables also may alter the degree of volatility partitioning and protein conformation in ways that cannot be adequately captured using a single protein docking model [6,16].

To address some of these limitations, recent advances in computational food science involve employing combinations of molecular docking and molecular dynamic (MD) simulations. Molecular dynamic simulations allow researchers to study the protein’s inherent flexibility, hydration shell interactions, and temperature-dependent conformational variability, as well as interactions with both the matrix and solvent, which are typically unaccounted for in rigid receptor docking simulations. Compared to both molecular-level predictive modeling and macroscopic flavor stability, the results generated from hybrid approaches represent a more realistic view of flavor stability mechanisms when calibrated to experimental kinetic data (volatile release profiles and structural spectroscopy) [75,76]. This mismatch is an indication that it is essential to factor in the effects of matrices in translating molecular prediction into real-life applications of food.

### 6.3. Outlook: Toward Docking-Informed Process Design

The strongest translational value of molecular docking, especially when combined with molecular dynamics simulations, offers the possibility that it will be used to inform early-stage process/formulation design as the basis of flavor-stable protein ingredients. Docking can be used to supplement the choice of the drying conditions and formulations that have higher chances of preserving preferred sensory qualities by producing relative binding rankings among protein sources, aroma compounds, and oxidative states [16].

In the case of novel/emerging protein sources where the empirical stability data are frequently scarce, docking-guided screening is a useful approach to minimize the need to conduct large-scale pilot experiments. Nevertheless, it is imperative to have strong experimental calibration. Predictions achieved in docking need to be verified with volatile fingerprinting (HS-GC-IMS or GC-MS), protein oxidation markers, and structural analyses before they can be considered a reliable tool to inform industrial decision-making [53,77]. Finally, the combination of molecular modeling, analytical methods, and process engineering creates a multi-scale link between the atomic-level interactions and the sensory results at the macro-scale. The method forms the basis of accuracy in dehydration methods that maximize flavor retention, energy efficiency, and sustainability in a wide variety of plant and marine protein structures [75].

## 7. Challenges, Standardization, and Future Perspectives

Although Section 3, Section 4, Section 5 and Section 6 illustrate the molecular mechanism and experimental evidence of advanced dehydration technologies and predictive modeling, three major challenges to the industrial implementation can be identified: scalability, standardization, and intelligent process control.

### 7.1. Scalability and Process Robustness

HPD and MVD are advanced drying technologies that work at the laboratory and pilot levels but have serious problems at the large-scale level during industrialization. Preventing case hardening in heterogeneous protein matrices requires precise HPD control with high precision in terms of humidity and airflow. MVD is confined by the depth of microwave penetration and the nonuniformity of fields, which may pose a risk of localized overheating and protein aggregation as well as oxidative damage. They can be solved using hybrid systems (e.g., MVD + airflow) and techno-economic models that have flavor retention measurements [16].

### 7.2. Structural Variability and Standardization of Multi-Source Protein Isolates

Sustainable protein systems rely on heterogeneous side-stream materials, which add variability in amino acid composition, acid oxidation history, lipid association, and extraction history, which influence conformation, lipid-binding capacity, and flavor-binding capacity. They do not have any functional benchmarks compared to standardized soy or dairy proteins, which makes these isolates hard to process reproducibly [23].

A suggested molecular standardization system might be based on the accepted measures: (1) Carbonyl Index in the form of DNPH assay (~25 nmol/mg level), (2) Surface Hydrophobicity in the form of ANS fluorescence, (3) Secondary Structure in the form of FTIR Amide I deconvolution (α-helix/β- sheet ratios), and (4) Docking-derived -G of major volatiles (e.g., hexanal, esters). These, together with HS-GC-IMS fingerprinting, allow real-time categorization of isolates before the dehydration process and less uncertainty in the process [44,78].

### 7.3. Intelligent Food Manufacturing and Real-Time Quality Control

AI and ML algorithms can combine docking simulations, oxidation marker data, and HS-GC-IMS fingerprint data to predict quality and adaptively produce products. For example, machine learning models comparing volatile compound profiles to near-real-time sensor information will allow for identifying the first signs of flavor decline, enabling dynamic adjustment of drying parameters (e.g., relative humidity) based on the risk of carbonyl formation [79].

A Digital Twin is a virtual representation of a production process, which includes a continuous update mechanism using the latest available spectroscopic and sensor data. The Digital Twin concept offers huge possibilities for simulating matrix influence on flavor retention at an industrial scale. Unlike current end-product inspections, this technology will be able to predict shelf-life via predictive models specifically applicable to clean label powder without artificial preservatives [7,80].

#### Concluding Remarks: Toward a Structure–Function Predictive Roadmap

The increasing demand for sustainable and diversified protein resources requires understanding how processing technologies, especially drying, influence protein structure and functionality. Drying is indispensable for stabilization, but it imposes complex physicochemical stresses that induce oxidative modifications and structural reorganization in protein matrices. These changes directly affect the exposure of hydrophobic residues, aggregation characteristics, and the availability of binding cavities that control the retention and release of volatile flavor compounds. This review has highlighted the important role of oxidative reactions and conformational dynamics in forming protein–flavor interactions during dehydration, identifying protein carbonylation and lipid–protein crosstalk as key drivers of binding-site disruption, flavor loss, and off-flavor formation.

By bringing together evidence across globular plant proteins, fibrous marine proteins, and hybrid plant–marine systems, we have proposed a structure–process–function framework that links intrinsic architecture, drying-induced structural and oxidative changes, and flavor-binding behavior. Within this framework, advanced drying technologies such as heat pump dehydration, microwave vacuum drying, and vacuum freeze-drying can be rationally positioned according to their ability to preserve native-like structural features, mitigate oxidation, and maintain reversible flavor-binding pockets. Computational tools, particularly molecular docking, supported by molecular dynamics simulations, offer an additional layer of insight by visualizing binding pockets and estimating how structural perturbations affect ligand affinity. When combined with experimental readouts such as HS-GC-IMS fingerprints, spectroscopic markers of secondary/tertiary structure, and quantitative oxidation indices, these approaches create a multiscale bridge from atomic-level interactions to macroscopic sensory performance.

Looking forward, the predictive design of flavor-stable protein ingredients will depend on the combination of this structure–process–function perspective with standardized molecular indicators and intelligent process control. Priority directions include establishing molecular benchmarks for heterogeneous protein isolates (e.g., carbonyl index, surface hydrophobicity, secondary-structure profiles, docking-derived binding energies), systematically linking these metrics to volatile profiles under different drying histories, and embedding such relationships into AI- and digital twin-driven drying control strategies. This is particularly critical for underexplored sustainable sources, such as tropical plant proteins and marine by-products, where structure–flavor relationships remain poorly characterized. Ultimately, a predictive, structure-aware approach to dehydration will enable the rational design of next-generation plant, marine, and hybrid protein systems with improved flavor stability, functional performance, and scalability, supporting the transition toward more sustainable and sensory-optimized food systems.

## Figures and Tables

**Table 1:** Impact of advanced drying technologies on the structural stability and flavor-retention profiles of diverse sustainable protein architectures.

No.	Protein System	Drying Technique	Structural Evidence	Oxidative Indicators	Flavor Interaction Mechanism	References
1	Peach Palm (Fruit protein)	Vacuum drying	Protein denaturation; limited aggregation	Moderate carbonyl increase; low SH loss	Enhanced volatile retention; reduced off-flavors	[14,46]
2	Soy Protein	Freeze-drying (VFD)	High retention of secondary structure; limited aggregation depending on moisture conditions	Low to moderate oxidation; preservation of sulfhydryl groups	Preserved hydrophobic regions—enhanced volatile retention	[47,48]
3	Soy Protein	Thermal drying	Increased β-sheet content and surface hydrophobicity	Oxidation and disulfide bond formation	Volatile formation via Maillard; off-flavor risk	[21,48,49]
4	Pea Protein	Freeze-drying (VFD)	Stabilized globular conformation; limited unfolding	Low ROS formation; preserved functional SH groups	Structural preservation—improved emulsification and volatile retention	[50,51]
5	Pea Protein	Thermal drying process	Partial denaturation and aggregation depending on drying severity	Increased carbonyl formation and sulfhydryl oxidation	Increased interaction with lipid-derived volatiles; potential off-flavor formation under oxidative conditions	[34]
6	Scallop Myofibrillar Protein	Vacuum freeze-drying (VFD)	Preservation of actin-myosin structure; reduced shrinkage and microstructural collapse	Minimal carbonyl formation; preserved protein integrity	Retains adsorption sites and limits release of fishy volatiles	[4,52]
7	Scallop Myofibrillar Protein	Heat pump drying (HPD)/hot air drying (HAD)	Protein denaturation; aggregation; structural contraction	Increased carbonyl content; sulfhydryl loss; ROS formation	Oxidation-induced aldehyde formation- increased off-flavor release compounds	[52]

Note: HPD, Heat Pump Dehydration; VFD, Vacuum Freeze-Drying; Values or observations are synthesized from representative studies cited in Section 3.

**Table 2:** Comparative quantitative data on drying-induced oxidative modifications and flavor-binding behavior across marine and plant protein systems.

Protein System	Processing Condition	Carbonyl Content (nmol/mg Protein)	Free SH (nmol/mg Protein)	α-Helix (%)	β-Sheet (%)	Volatile Retention/ Binding	References
Fish myofibrillar protein (fresh)	Control (undried)	0.37	-	Highest (baseline)	Lowest (baseline)	-	[32]
Fish myofibrillar protein (fresh)	Hot air drying	2.17 (+487%)	-	Significant decrease	Significant increase	-	[32]
Fish myofibrillar protein (fresh)	Heat pump drying	1.04 (+181%)	-	Partially preserved	Moderately increased	-	[32]
Fish myofibrillar protein (fresh)	Vacuum freeze-drying	0.52 (+41%)	-	Best preserved	Least increased	-	[32]
Sturgeon myofibrillar protein	Thermal 50 °C	-	-	13.60	38.16	-	[56]
Sturgeon myofibrillar protein	Thermal 70 °C	-	-	11.71	41.90	-	[56]
Sturgeon myofibrillar protein + MDA	Thermal 70 °C	-	-	11.69	45.49	-	[56]
Sturgeon myofibrillar protein + HNE	Thermal 70 °C	-	-	10.98	43.99	-	[56]
*M. rosenbergii* myofibrillar protein	MVD- initial stage (M85)	Baseline	Baseline (T-SH and F-SH highest)	-	-	-	[5]
*M. rosenbergii* myofibrillar protein	MVD -final stage (M10)	+2.00 nmol/mg vs. M85	T-SH −76.47%; F-SH −41.23% vs. M85	-	-	-	[5]
Soy protein isolate (SPI)	Chemical oxidation (AAPH, control)	5.78	-	Decreased with oxidation	-	-	[57]
Pea protein isolate (PPI)	Native dispersion (flavor binding)	-	-	-	-	K hexanal ≈ 684 M^−1^; K (E)-2-octenal ≈ 8208 M^−1^	[58]
Chickpea, pea, soy, fava bean, whey	Native dispersions (partitioning mode	-	-	-	-	RHC methyl octanoate: 40%→10%; decanone RHC: 4.5–51%→0.7–9.1%	[59]

Note: -, data not reported or not applicable for the cited study. MDA, malondialdehyde; HNE, 4-hydroxy-2-nonenal; MVD, microwave vacuum drying; RHC, relative headspace concentration; K, binding association constant; T-SH, total sulfhydryl; F-SH, free sulfhydryl;. Carbonyl content for fish myofibrillar protein is expressed as nmol/g protein. Sturgeon MP secondary structure values expressed as a percentage of total secondary structure. M85 and M10 refer to moisture content stages during MVD.

**Table 3:** Molecular markers and analytical methodologies for assessing drying-induced protein oxidation and conformational shifts in relation to flavor-binding functionality.

Oxidation Marker/Technique	Structural Effect	Functional Impact	Measurement	Relevance to Flavor Binding	References
Protein carbonyls (DNPH assay)	Protein unfolding, aggregation	Decreased solubility, emulsifying capacity, and gelation	nmol carbonyls per mg protein	Increased volatile release (reduced retention)	[23,53]
FTIR spectroscopy	Decreased α-helix; increased β-sheet	Dose-dependent changes in gel strength (mild oxidation: increased gel strength; severe oxidation: decreased functionality)	Amide I band shifts	“Pocket occlusion” of hydrophobic sites (reduced accessibility of flavor-binding regions)	[23,56]
Intrinsic fluorescence	Tryptophan burial; changes in tertiary structure	Decreased water-holding capacity and emulsifying activity	λ_max shift	Changes in surface hydrophobicity	[56]
Matrix effects (pH, ionic strength, lipids)	Conformational modulation of proteins	Variable effects (for example, increased aggregation in marine myofibrillar protein)	Spectroscopic measurements combined with function assays	Altered volatile partitioning and retention	[6,16]

Note: DNPH, 2,4-Dinitrophenylhydrazine (carbonyl assay); FTIR, Fourier-Transform Infrared Spectroscopy; λ_max, wavelength of maximum fluorescence emission. Structural transitions refer to changes in secondary (α-helix, β-sheet) and tertiary structure that modulate the accessibility of flavor-binding pockets.

**Table 4:** Representative molecular docking studies of protein-flavor/ligand interactions relevant to food processing systems.

Protein Systems	Flavor Compound	Binding Energy (ΔG, kcal/mol)	Key Interaction Forces	Validation Method	Software	References
Soy protein isolate	Hexanal (beany off-flavor)	−4.5 kcal/mol (hydrophobic cavity)	Hydrophobic interactions, H-bondig	HS-SPME-GC-MS, circular dichroism	AutoDock	[63]
Pea protein isolate (PPI)	Hexanal, (E)-2-octenal	−11.4 kcal/mol (hexanal), −8.3 kcal/mol (*E*)-2-octenal)	H-bonding (hexanal); hydrophobic (2-octenal)	Headspace GC/MS, fluorescence	AutoDock Vina	[58]
Myofibrillar protein (myosin, PDB:3QMA)	Malondialdehyde (MDA)	−5.5 kcal/mol	Hydrogen bonds (6 bonds, 1.8–3.5 Å)	ITC (ΔG = −25 to −29.8 kcal/mol), FTIR, fluorescence	AutoDock Vina	[56]
Myofibrillar protein (myosin, PDB: 3QMA)	4-Hydroxy-2-nonenal (HNE)	−4.1 kcal/mol	Hydrogen bonds (3 bonds, 3.0–3.2 Å)	ITC (ΔG = −74.3 to −78.9 kcal/mol), FTIR, fluorescence	AutoDock Vina	[56]
T1R1/T1R3 umami receptor (homology model, Atlantic cod myosin)	INKPGL (umami hexapeptide)	−109.1 kcal/mol (CDOCKER energy)	H-bonds, electrostatic, hydrophobic (S66, S104, S146, S147, E45, E47)	E-tongue + sensory evaluation	Discovery Studio (v17.2.016349) (CDOCKER)	[65]

ΔG = Gibbs free energy of binding; more negative values indicate stronger predicted binding affinity. Where quantitative binding energy values were not explicitly reported, qualitative interaction profiles are described. Native vs. oxidized comparisons remain limited in the current literature.

## Data Availability

No new data were created or analyzed in this study. Data sharing is not applicable.
